# Demographic changes of hepatitis B virus infection in Iran for the last two decades 

**Published:** 2017

**Authors:** Hamid Mohaghegh Shelmani, Peter Karayiannis, Sara Ashtari, Mohammad Amin Mahmanzar, Binazir Khanabadi, Niusha Modami, Fatemeh Gholipour, Fatemeh Zare, Mohammad Reza Zali

**Affiliations:** 1 *Gastroenterology and Liver Diseases Research Center, Research Institute for Gastroenterology and Liver Diseases, Shahid Beheshti University of Medical Sciences, Tehran, Iran.*; 2 *University of Nicosia Medical School, Nicosia, Cyprus. *; 3 *Basic and Molecular Epidemiology of Gastrointestinal Disorders Research Center, Research Institute for Gastroenterology and Liver Diseases, Shahid Beheshti University of Medical Sciences, Tehran, Iran.*; 4 *Foodborne and Waterborne Diseases Research Center, Research Institute for Gastroenterology and Liver Diseases, Shahid Beheshti University of Medical Sciences, Tehran, Iran *

**Keywords:** Hepatitis B virus, hepatitis B surface antigen (HBsAg), Epidemiology, Vaccination, Iran

## Abstract

**Aim:**

The objective of this study was to evaluate the impact of the hepatitis B virus (HBV) vaccination program, 24 years after its implementation, by analyzing patients with hepatitis B surface antigen (HBsAg) infection based on gender and age group.

**Background:**

Since the launch of the first universal vaccination program against HBV in Iran in 1993, the epidemiological pattern of HBV prevalence may have changed in our country.

**Methods:**

All data for this cross-sectional study were collected from medical records of HBsAg positive patients, who were referred to the Golhak and Armin private laboratories and also to the Gastrointestinal Department of Tehran’s Taleghani Hospital and Day Hospital in Iran over a period of 5 years (2011-2016). In total, 8,606 HBsAg positive subjects were assessed according to gender and age group.

**Results:**

The rates of HBsAg carriage were 0.8%, 7.8%, 49.3%, 27.9% and 14.1% among subjects under 14 years old, 15-24 years, 25-44 years, 45-59 years and those older than 60 years, respectively. According to the age subgroup analyses; the highest (26.2%) and lowest (0.6%) rate of HBsAg positivity was seen in the 31-40 age group and younger than 10 year old children, respectively.

**Conclusion:**

Global vaccination against hepatitis B has significantly reduced carrier rates among children and teenagers under 20 years old in this country. Nevertheless, HBsAg carriers still remain highly prevalent among 25-35-year age group. Therefore, the decline is limited to the younger population born after 1993, and it remains high in the middle-aged individuals.

## Introduction

 In spite of an effective vaccine against hepatitis B virus (HBV), the infection remains a major global health problem. It can cause chronic hepatitis, cirrhosis and hepatocellular carcinoma (HCC) and is associated with significant morbidity and mortality (1-4). According to the World Health Organization (WHO) about 2 billion people have been infected by HBV, among whom more than 350 million people worldwide are chronically infected being hepatitis B surface antigen (HBsAg) positive (5, 6). The global prevalence of HBV varies geographically; from< 2% in low endemic areas of Western Europe, North America and Australia, 2%-7% in intermediate endemic areas such as Southern and Eastern Europe, to more than 8% in high endemic areas in Africa and Asia (7). According to WHO (8) and CDC (9) Iran and Middle Eastern countries are classified as intermediate risk region with a prevalence of 2% to 7%; approximately two million Iranian adults are chronically infected (10-12). 

The HBV National Vaccination Program has been included in the extended program of immunization (EPI) for all newborns and high risk groups since 1993 in Iran (13). Because of the vaccination program, the epidemiological pattern of HBV prevalence is likely to have changed over time in our country. The reduction in acute and chronic HBV infection due to the implementation of the HBV National Vaccination Program has already been reported (12, 14, 15). Two sero-epidemiologic studies were conducted by Zali et al. (16) before and after the mass vaccination against hepatitis B in 1993 on a representative sample of 1/1000 of the population of Iran. The overall prevalence of chronic HBV infection was reported as 1.7% in the 1990s. In addition, among children aged 2 to 14 years, the prevalence of HBsAg carriers decreased significantly (1.3% vs. 0.8%, P<0.05) between 1991 and 1999. According to this study, the overall prevalence of HBsAg declined within 6 years of the implementation of the hepatitis B vaccination program in neonates in 1993, which appears to be mainly due to the vaccination of people born after 1993.

After 24 years of implementation of the HBV vaccination in Iran, the epidemiology of HBV infection in Iran has been changed and neonatal vaccination with high coverage is the main cause of this change (17). In spite of this success, hepatitis B remains an important cause of morbidity and mortality in adults. Therefore, to investigate the epidemiological changes of hepatitis B in the country after two decades of vaccination, the present study was conducted and its results may prove useful in establishing public health policies against HBV infection in the Islamic Republic of Iran. 

## Methods

All data for this cross-sectional study were collected from medical records of HBsAg positive patients, who were referred to the Golhak and Armin private laboratories and also to the Gastrointestinal Department of Tehran’s Taleghani Hospital and Day Hospital in Iran over a period of 5 years from 2011 to 2016. In total, 8,606 HBsAg positive subjects were assessed according to gender and age group. The patients were divided into five age groups (≤14, 15-24, 25-44, 45-59, and ≥60 years). The main reason of this category was to divide patients based on the year of their birth before and after 1993 and in order to get more information, patients were divided into subgroups. To compare the results of this study with a previous study (16) and examining the epidemiological changes of hepatitis B in different age groups, patients were also divided into the same 4 age groups as the previous study (≤14, 15-39, 40-69 and ≥70 years). The study was approved by the Ethics Committee of the Research Institute for Gastroenterology and Liver Diseases, Shahid Beheshti University of Medical Sciences, Tehran, Iran.

**Statistical Analysis**

Data were entered and analyzed using the statistical package for social sciences (SPSS) for windows version 21 software (SPSS Inc., Chicago, IL, USA). Descriptive statistics and frequency distribution such as mean, standard deviation and percentage were employed. A chi-square test was used to compare the qualitative variables. P<0.05 was considered as statistically significant.

## Results

A total of 8,606 HBsAg positive patients with a mean age of 42±14.8 (± standard deviation) years old were enrolled in this cross-sectional study and 4939 (57.4%) patients were male. The age range of patients were 2-97 years. Table 1 presents the baseline clinical characteristics of the 8,606 participants. All 8,606 HBsAg positive subjects were collected over a period of 5 years (2011-2016). The numbers of HBsAg carriers were 72 (0.8%), 669 (7.8%), 4246 (49.3%), 2405 (27.9%) and 1214 (14.1%) among subjects under 14 years, 15 to 24 years, 25 to 44 years, 45 to 59 years and older than 60 years, respectively. 

**Table 1:** Baseline characteristics of hepatitis B surface antigen (HBsAg) among the study participants from 2011 to 2016

Characteristic	Years						Total
	2011	2012	2013	2014	2015	2016	
No of patients	970 (11.3)	1105 (12.8)	1340 (15.6)	1385 (16.1)	1680 (19.5)	2126 (24.7)	8606 (100)
Age (mean±SD)	40.6±15.1	40.1±14.5	38±14.4	42.9±15.2	42.5±15.3	40.0±14.0	42.5±14.8
Male	588 (11.9)	641 (13.0)	790 (16.0)	772 (15.6)	964 (19.5)	1184 (24.0)	4939 (100)
Female	382 (10.4)	464 (12.7)	550 (15.0)	613 (16.7)	716 (19.5)	942 (25.7)	3667 (100)
Age group							
≤14	11 (15.3)	9 (12.5)	20 (27.8)	7 (9.7)	15 (20.8)	10 (13.9)	72 (100)
15-24	112 (16.7)	119 (17.8)	138 (20.6)	70 (10.5)	117 (17.5)	113 (16.9)	669 (100)
25-44	479 (11.3)	576 (13.6)	685 (16.1)	604 (14.2)	826 (19.5)	1076 (25.3)	4246 (100)
45-59	240 (10.0)	279 (11.6)	345 (14.3)	471 (19.6)	461 (19.2)	609 (25.3)	2405 (100)
≥60	128 (10.5)	122 (10.0)	152 (12.5)	233 (19.2)	261 (21.5)	318 (26.2)	1214 (100)

**Figure 1:**
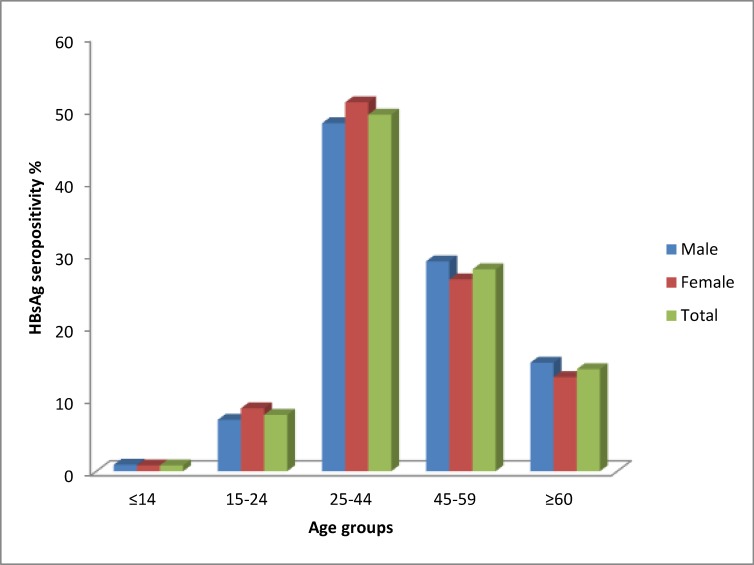
Prevalence of HBsAg among the study participants by age group and gender

**Figure 2:**
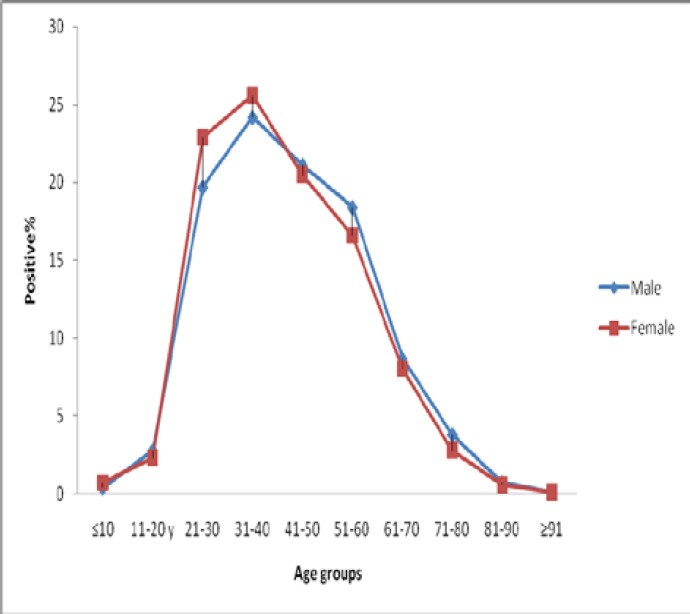
Comparison of hepatitis B surface antigen (HBsAg) among the study participants by gender

**Figure 3:**
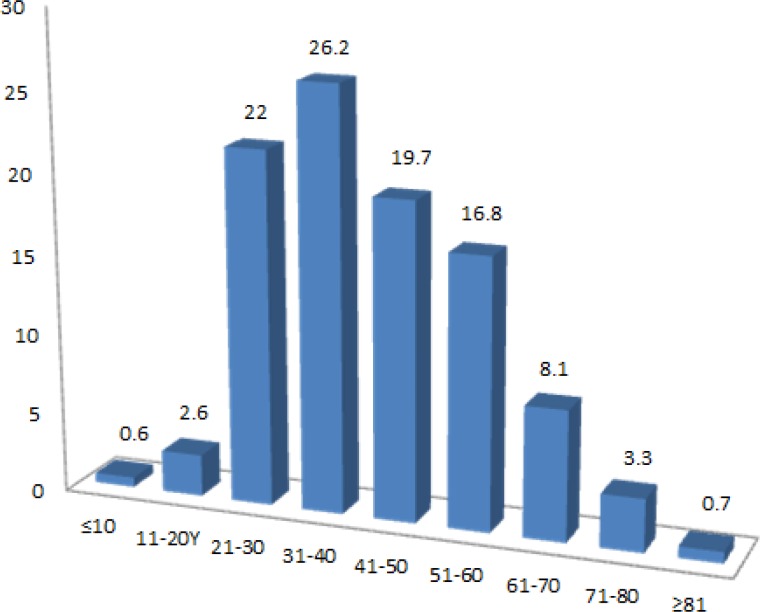
Comparison of hepatitis B surface antigen (HBsAg) among the study participants by age groups

**Figure 4:**
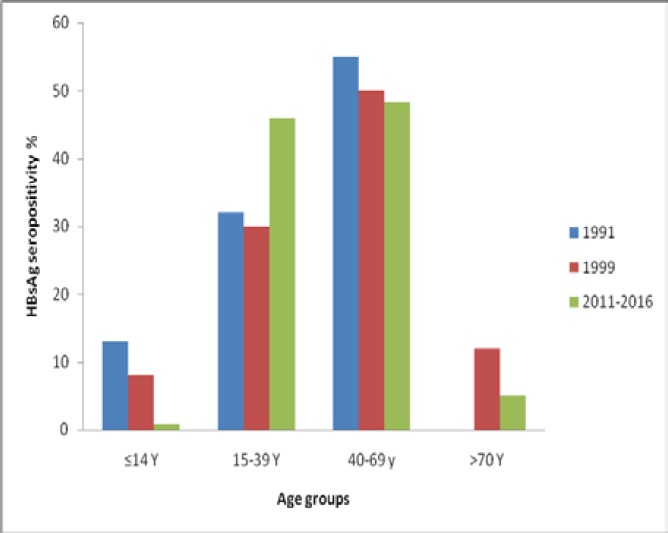
Comparison of hepatitis B surface antigen (HBsAg) patients in 1991, 1999 and 2016

The prevalence of HBsAg among the study participants by age group and gender is shown in Figure 1. The highest (49.3%) and lowest (0.8%) rate of HBsAg positivity were seen in the 25 to 44 age group and the patients youmger than 14 years old, respectively. According to gender, the HBsAg prevalence rates for females among 15-24 and 25-44 were significantly higher than males (P<0.05). According to the age subgroups analyses; among subjects aged 21-30 and 31-40 years, the HBsAg prevalence rates for females were 22.9% and 25.6%, respectively, which are significantly higher than the rates for males (19.7% and 24.2%, respectively) (P<0.05). However, among subjects aged 51 to 60 years, HBsAg prevalence rates for males were significantly higher than the rates for females (18.4% vs. 16.6%, P<0.05). There was no significant difference between the HBsAg prevalence in males and females in other age groups (Figure 2).

When dividing the age groups into smaller groups as subgroups, the highest (26.2%) and lowest (0.6%) rate of HBsAg positivity were seen in 31-40 age groups and younger than 10 year old children, respectively. Figure 3 presents the HBsAg positivity among the study participants by subgroups.

## Discussion

This study provided an up-to-date assessment of hepatitis B infection according to the gender and age among HBsAg positive patients in Tehran during the period of 2011-2016. To our knowledge, this is the largest community-based study ever conducted in Iran after two decades of vaccination against HBV.

Actually, in 1992 the World Health Organization (WHO) recommended global vaccination against HBV. The hepatitis B vaccination program in Iran has been made available to all newborns and high risk groups such as healthcare workers since 1993. An extended vaccination program for adolescents born during 1989-1992 was performed during 2007-2010 (13). The past 24 years, all newborns have been covered by the program and have received the vaccine in three stages over regular periods; at birth, at 1.5 months and nine months after birth (18, 19). 

In order to evaluate the impact of the hepatitis B vaccination program over the last two decades, we grouped the study participants into five groups with the cut-off of age set as ≤14, 15-24, 25-44, 45-59 and over 60 years old. Thus, we generated five birth cohorts for analysis: participants born during 2002-2014, 1992 to 2001, 1972 to 1991, 1957 to 1971 and before 1956. This study showed that children aged younger than 14 years old and young adults aged 15-24 years old had the lowest rate of HBsAg positivity (0.8% and 7.8%, respectively) compared with the other groups. The greatest effect of hepatitis B vaccination was observed in the less than 20 years old age groups. This transformation occurred after the hepatitis B vaccine was recommended as part of routine infant immunization 24 years ago (1993), which shifted children to a relatively low-risk group. In other words, national vaccination of Iranian neonates has significantly decreased the carrier rate among young children and the average age of the infected individuals has increased. It seems that the root of HBV transmission in Iran is changing from vertical to horizontal in recent years (15, 20). However, since the past few years, all pregnant women in Iran have been required by law to be checked for HBV and in case a mother found to be an HBV carrier, adequate instructions are given to inject her newborn with hepatitis B immunoglobulin (HBIG) immediately after delivery (10).

The highest rate of HBsAg occurred in adults aged 25 to 59. According to the age subgroup analyses, the HBsAg prevalence of (27.6%) was highest among adults aged 25-35 years old. Patients aged 25 to 35 years were born before 1993, so based on national HBV vaccination program they were vaccinated during 2007 to 2010 when they were 18 to 28 years old or most of them may have not received the vaccine at all. Hence, the effectiveness of hepatitis B vaccination in adults is lower than in children. The vaccination against HBV before the first year of life is more effective than after that (21). On the other hand, previous studies in Iran showed that injection drug users, prisoners, drivers and healthcare workers had the greatest risk of becoming HBsAg positive (22-24). In addition, high-risk behaviors such as having multiple sexual partners, tattoos and cupping can increase the likelihood of hepatitis B infection. Unfortunately, these behaviors are more evident in the young population and they can be the reasons for the high rates of HBsAg in this age group. Therefore, with the exception of national vaccination program, measures should be taken to focus on the preventative measures high risk populations and elimination of such high risk behaviors. Iran has been conducting the vaccination program for high-risk groups, such as medical staff and clinical students (25). Systematic reviews and meta-analysis studies have reported the rate of HBV vaccination coverage 70.1%, 73%, 72.2% and 90.9% for physicians, nurses, dentists and healthcare personnel in Iran, respectively (26-29). 

The findings in this study indicated that there was a significant decline in hepatitis B epidemics among children (≤14 years old) and also in patients older than 70 years when compared with previous reports; (8% in 1999 vs. 0.8% in 2016) and (12% in 1999 vs. 5% in 2016), respectively (16). The comparison between the results of the present study and a previous report by Zali et al. is shown in Figure 4. The prevalence of HBsAg positivity in individuals aged 15-39 years old was significantly higher than previous studies. That can be due to the horizontal mode of transmission which is more important than the vertical one in Iran.

We had some limitations in this study. The data from this study were collected from medical records of the patients; thus, we did not have access to information such as family history or risk factors in these patients.

In conclusion, global vaccination against hepatitis B has significantly reduced carrier rates among children and teenagers under 20 years old in this country. Nevertheless, it seems to be highly prevalent among HBsAg carriers aged 25 to 35 years. Therefore, the decline is limited to the younger population born after 1993, and viral persistence remains in the middle-aged population. These efforts should be maintained in order to make a greater progress in the future. To achieve the national goal of decreasing the prevalence of HBsAg of the entire population by the ideal vaccine schedule to protect both infants and adolescents, intensifying vaccination of high-risk groups and control on interfamily transmission is essential. Moreover, screening pregnant women for HBV infection, and follow-up of babies of the HBsAg positive mothers is recommended.
